# Annotation of 200 Insect Genomes with BRAKER for Consistent Comparisons across Species

**DOI:** 10.1038/s41597-026-06840-0

**Published:** 2026-02-19

**Authors:** Stepan Saenko, Katharina J. Hoff, Mario Stanke

**Affiliations:** 1 Institute of Mathematics and Computer Science, University of Greifswald, Greifswald, 17489 Germany; 2 Center for Functional Genomics of Microbes, University of Greifswald, Greifswald, 17489 Germany

**Keywords:** Sequence annotation, Genome informatics

## Abstract

The annotation of genomes progresses slower than their sequencing and assembly. Also, species that were previously annotated can benefit from reannotation using more recent RNA-Seq and protein data, as well as from state-of-the-art annotation methods whose accuracy has improved. Heterogeneous annotations performed with different tools and protein databases can introduce artifactual differences when comparing gene sets or gene structures between species. Recently, the BRAKER3 annotation pipeline was introduced that integrates evidence from RNA-Seq and a protein database. Here, we introduce an automated genome annotation workflow based on BRAKER3 that allows one to annotate a list of species with minimal manual intervention. We selected a diverse set of 200 insect species from different families, including 85 species previously lacking annotations in GenBank. Using currently available RNA-Seq and protein sequence data, we applied our automated workflow to annotate these genomes and conducted downstream analyses typically performed in comparative genomics studies. We present the resulting gene structures, protein sequences, gene ontology terms, orthologous gene groups and a species tree.

## Background & Summary

Over the past two decades, the number of sequenced insect genomes has increased dramatically, from just twenty species 20 years ago to over 4,000 today, according to statistics from GenBank and NCBI datasets^1,2^.

Additionally, other large-scale initiatives and external databases, such as Tree of Life^3^, contribute to this growing pool of genomic data. However, having access to raw data is only the first step; accurate and comprehensive genome annotations are essential to address key biological questions and challenges in phylogenetics, comparative genomics, evolutionary biology, the analysis of gene functions and the study of developmental pathways.

**Fig. 1:**
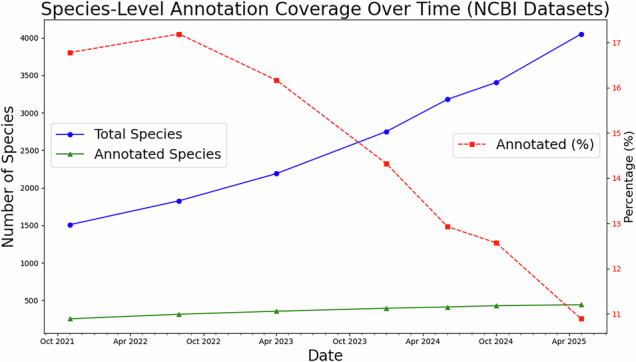
Total number of insect species in NCBI Datasets with an assembly at least at scaffold-level and the percentage of species where at least one assembly has an annotation in NCBI Datasets.

While the number of insect species with scaffold- or chromosome-level assemblies in GenBank has risen to 3,062 the percentage of species with annotations in GenBank has decreased to only 10%, underscoring a significant annotation bottleneck. Similarly, according to the independently maintained NCBI Datasets resource, there are 3,995 insect species with scaffold- or chromosome-level assemblies, of which approximately 11% have annotations. These two numbers differ because GenBank and NCBI Datasets follow different update schedules and inclusion criteria, but both datasets consistently highlight the same trend: annotation coverage has not kept pace with the rapid increase in newly assembled genomes (Fig. 1).

To maximize the benefit of genomic data, annotation pipelines must meet several criteria: algorithms must be up-to-date, raw data should be accessible, and the entire process must be reproducible; additionally, annotations must be high quality, standardized, and comparable between species.

Comparability, in this context, means that observed genomic differences, such as gene truncations, the presence or absence of exons or even whole genes, or variations in gene structure, should reflect true biological distinctions rather than errors or missing genes or splice forms introduced by differing annotation methods or the use of other data sets (e.g., proteins or RNA-Seq).

Algorithm development has recently been shown to lead to improved accuracy^4–6^, in addition to the progress that can be achieved when applying old algorithms to additional data. For example, the average increases in F1 scores when identifying complete protein-coding transcripts in eleven species were 4 percent points from BRAKER1 to BRAKER2 and an additional 24 percent points from BRAKER2 to BRAKER3. The latter achieved an average transcript-level F1 score of about 60%.

Unfortunately, the current state of eukaryotic genome annotation is still not precise enough to ensure that all, or even most, of the structural differences identified between the annotations of closely related species can be reliably interpreted as biological variations. In addition, the problem of false positive differences can be exacerbated when annotations are generated by a variety of methods. For example, such heterogeneity in annotation methods has been shown to dramatically increase the apparent number of lineage-specific genes^7^.

BRAKER3^5^ integrates evidence from RNA-Seq in addition to evidence from a protein database, which was already integrated by BRAKER2^8^. In both these run modes, the BRAKER pipeline automatically trains the parameters of hidden Markov models using the genome and the provided evidence and predicts protein-coding genes using the evidence again, including alternative splice forms. BRAKER3 was benchmarked under controlled conditions on the similarity between database and target proteins on 11 species, including *Drosophila melanogaster*, *Bombus terrestris* and *Parasteatoda tepidariorum*. It performed best among the pipelines compared^5^.

The BRAKER pipeline can be started with a single command line and, contrary to MAKER^9^, for example, does not require manual steps. However, the RNA-Seq data and a protein database have to be provided by a user and the input genome should be repeat masked. In addition, it is advisable to perform quality control steps after annotation, such as the execution of BUSCO^10^ and OMArk^11^. As such preprocessing and postprocessing steps to annotation can require substantial manual effort if done for a large number of species, we have developed an automated *VARUS-BRAKER workflow* that performs them automatically and integrates multiple tools: VARUS^12^, which automatically collects, selects, and aligns RNA-Seq data; repeat masking; BRAKER2^8^ and BRAKER3^5^, as well as downstream tools for quality control. As a result, the new VARUS-BRAKER workflow used in this study requires minimal manual effort per genome. In its user-friendliest run mode, only the binomial names of the species need to be input.

For the insect annotation data presented here, we focused on Holometabola and a diverse set of their outgroups, as our goal was the study of evolutionary innovations along the branch leading to the most recent ancestor of Holometabola. However, the annotation data and the orthologous groups of genes presented here can serve as a foundation for diverse other studies of insect genomics^13^.

Holometabolous insects exhibit a unique larval stage that is morphologically distinct from the adult form. They undergo a complex transformation, with extensive reorganization and dedifferentiation of larval organs during the prepupal and pupal stages^14^. In contrast, hemimetabolous insects undergo a more gradual developmental process: their embryogenesis produces an adult-like larva that transitions into the adult form through a series of molts, with wings and genitalia typically appearing in the final molt. The origin of complete metamorphosis dates back approximately 400 million years^15^ to the early Devonian period, an era marked by significant evolutionary innovations, including the emergence of winged insects (Pterygota), the advent of insect flight, and the development of holometaboly – an adaptation that likely contributed to the evolutionary success of these insects.

We here present whole-genome annotations of the protein-coding genes of 200 insect species, 85 of which did not have annotations in GenBank. Gene structures were predicted with BRAKER. RNA-Seq data was integrated for all species where it was available as well as evidence from protein homology. All 4,259,838 identified proteins were functionally annotated with GO terms. We identified groups of orthologs in the whole set of 200 proteomes, and subsequently constructed multiple sequence alignments of these protein families and a maximum likelihood species tree.

## Methods

The primary goal of this study was to address the gap in insect genome annotations by employing state-of-the-art tools, ensuring consistent annotation quality. Using the BRAKER3 pipeline^5^, our objective was to minimize artifacts arising from heterogeneous annotation methods and incorporate the most current data, including protein and RNA-Seq datasets. To highlight the need for updates, we assessed the age of existing annotations using metadata from the NCBI RefSeq database, which provides reliable timestamps. Our analysis revealed a wide range of submission dates, with many annotations dating back several years. Specifically, out of the dataset examined, approximately 75% were submitted prior to 2022.

**Fig. 2:**
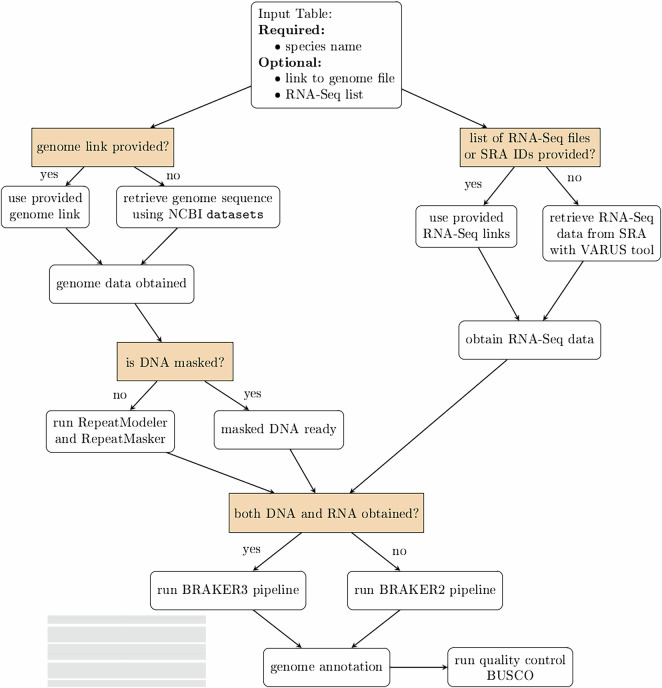
The scheme of the VARUS-BRAKER workflow.

The genome annotations presented in this work were generated using publicly available genome, RNA-Seq, and protein datasets. The analysis was carried out in three key stages: input specification, structural genome annotation, and downstream processing. An overview of the analysis workflow is shown in Fig. 2.

In the first stage, *input specification*, the user prepares a text table with pointers to the genomes and optionally to the RNA-Seq data. Each line in the table specifies one genome to be annotated. There are several ways to specify the input data. As a minimum, when genome and RNA-Seq data are accessible from Genbank and Sequence Read Archive (SRA, https://www.ncbi.nlm.nih.gov/sra/), it is sufficient if a row just contains the species name, e.g. *Apis mellifera*. In general, the table has the following columns: species name (mandatory), optional link to the genome file (file path or URL) and optional list of links for RNA-Seq data or a list of SRA IDs. If there are no genome data provided, datasets from NCBI (v14.16.0)^16^ will be used to download the assembly from GenBank, giving preference to assemblies tagged ‘reference’.

The second stage, *structural genome annotation*, encompasses all automatic steps up to and including the gene prediction. Initially, RepeatModeler2 (v2.0.4)^17^ and RepeatMasker (v4.1.4)^18^ generate a species-specific repeat library and soft-mask the genome. If no specific RNA-Seq data was specified, VARUS (v1.0.0) is used to automatically obtain RNA-Seq evidence. Note that for some insect species there are thousands of RNA sequencing runs deposited at SRA. VARUS uses NCBI’s fastq-dump to retrieve reads from SRA. VARUS incrementally downloads relatively small random read samples (e.g. 50 k–200 k reads per iteration) from a potentially large number of sequencing runs. The total amount of downloaded data and the sampling granularity can be controlled by user-defined parameters, such as the maximum number of batches and the batch size. The RNA-Seq reads are then aligned to the genome with HISAT2^19^. The alignments are used to detect libraries of poor quality and to prioritize the further sampling from libraries that complement the expression observed so far. For details and benchmarks, we refer to^12^. The resulting SAM files are converted to BAM format, merged if multiple libraries were used, sorted, and indexed using SAMtools^20^.

To obtain data to exploit homology with known proteins, the workflow automatically determines a suitable section of OrthoDB v12^21^ from the species name using NCBI’s Taxonomy (https://www.ncbi.nlm.nih.gov/taxonomy/). This protein database is downloaded, unless already present, from https://bioinf.uni-greifswald.de/bioinf/partitioned_odb12. Then the gene models are called by the BRAKER pipeline (v3.0.3), which itself entails steps to train GeneMark-ETP (v1.0.0)^22^ and AUGUSTUS (v3.5.0)^23^ and predict evidence-based gene structures with them. If only RNA-Seq data is available, the BRAKER3 mode is used, otherwise the BRAKER2 mode is used to predict protein-coding gene structures from the soft-masked genome. The BRAKER3 mode requires RNA-Seq and is preferred as it was benchmarked to be significantly more accurate than BRAKER2^5^. In BRAKER3 mode, it utilizes GeneMark-ETP^22^, which processes RNA-Seq alignments through StringTie2 (v2.2.1)^24^ to assemble transcripts. GeneMarkS-T (v4.30) screens the assembled transcripts for potential genes. Additionally, DIAMOND (v0.9.24.125)^25^ and GeneMark-EP+’s protein evidence pipeline are used to filter genes. GeneMark-ETP performs gene predictions based on self-training. Finally, AUGUSTUS is being trained on a reliable subset of predicted genes, and the final gene set is being consolidated using TSEBRA (v1.1.0)^26^. Protein evidence was incorporated during annotation, regardless whether BRAKER3 mode or BRAKER2 mode was used. This mode involves GeneMark-EP+(v4.72), which self-trained GeneMark-ES (v4.72) to identify seed gene sequences. These sequences are then aligned to the protein database using DIAMOND, followed by accurate spliced alignment using Spaln2 (v2.4.13g)^27^. The intermediate gene set generated by GeneMark-EP+ based on protein evidence was refined with AUGUSTUS.

Benchmark results comparisons to BRAKER3, when run with manually selected whole RNA-Seq libraries, are available in Section Technical Validation. Based on the experimental results, we chose a VARUS batch size of 75,000 reads and the maximum of 600 batches. Increasing these parameters did not improve accuracy or precision; it only increased processing time. The optimal VARUS runtime was less than four hours.

In the third and final stage, *downstream processing*, the workflow performs quality assessment and functional annotation. BUSCO^10^ is used to assess in particular the completeness of the predicted proteomes with regards to universal single-copy genes. Separately from the main workflow, we also conducted an additional analysis using FANTASIA^28^ to enhance functional annotation by integrating gene ontology (GO) terms and protein domain information, thereby improving both the accuracy and completeness of annotations.

### Species Selection

**Fig. 3:**
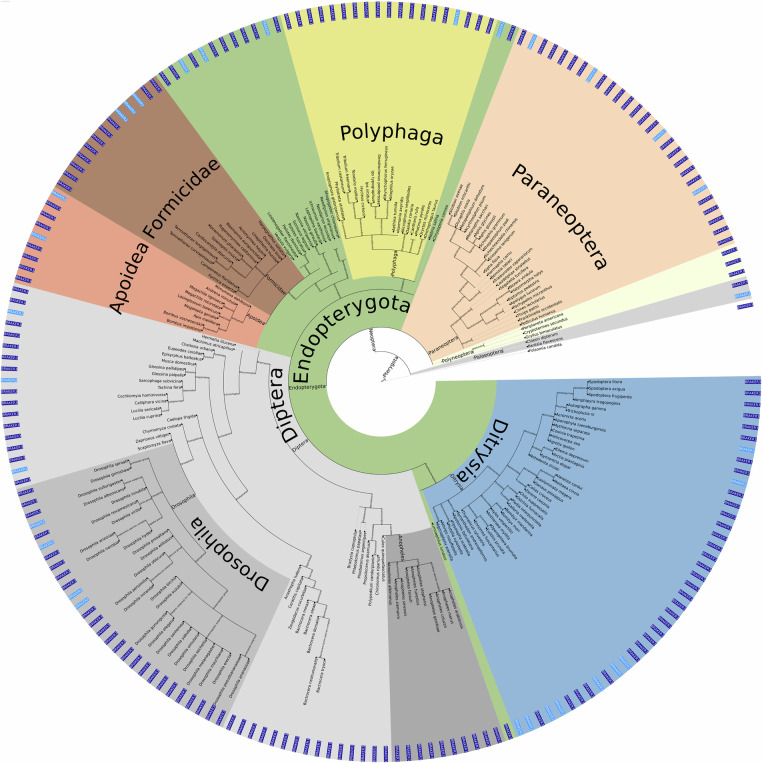
Taxonomic placement of the 200 selected insect species based on the NCBI taxonomy. Coloured sectors indicate major insect clades: *Polyphaga* (yellow), *Paraneoptera* (orange), *Diptera* (light gray), *Drosophila* (dark gray), *Ditrysia* (blue), and *Apoidea/Formicidae* (brown), with Endopterygota highlighted in green in the inner ring. A bright or dark blue label at the outer rim marks whether annotations performed in this study were done with BRAKER2 or BRAKER3, respectively.

As basis for selecting a sample of species, we used a tree derived from NCBI’s Taxonomy (Fig. 3). We used the NCBITaxa module from the ete3 package^29^ to handle taxonomy data, which included all insect species from the table at https://ftp.ncbi.nlm.nih.gov/genomes/GENOME_REPORTS/eukaryotes.txt with an assembly level higher than “contig”, resulting in a set of 3,062 species. We aimed to represent much of the insect diversity given our computational limit of roughly 200 species, and therefore selected species across the major insect clades (e.g. *Polyphaga*, *Paraneoptera*, *Diptera* including *Drosophila*, *Ditrysia*, *Apoidea* and *Formicidae*), as illustrated in Fig. 3.

First, a subset of species was manually selected. The remaining species were then automatically selected to maximize diversity. Manual selection used two criteria: the number of citations in Google Scholar, reflecting their scientific relevance, and the availability of RNA-Seq data in the Sequence Read Archive (SRA), ensuring sufficient transcriptomic evidence for robust annotation. To obtain the remaining species, we developed and run a custom script that identifies for a given tree, set of manually selected leaves *A* and a given total number *n* a set *S* of leaves such that *A* and *S* together contain *n* leaves, and the tree restricted to *S* ∪ *A* and its ancestors has maximum total branch length, where the length of a branch is the number of taxonomic levels it spans. This script maxSubtreeSet.pl is included in the VARUS-BRAKER repository on GitHub. This approach maximized diversity while including species that are suitable for comparative benchmarking or that are otherwise important. The choice also strikes a balance between reannotations (115) and annotations of previously unannotated genomes (85). Species with available RNA-Seq (169) were preferred over species without (31), as the accuracy of BRAKER is much higher in the former than in the latter^5^.

**Fig. 4:**
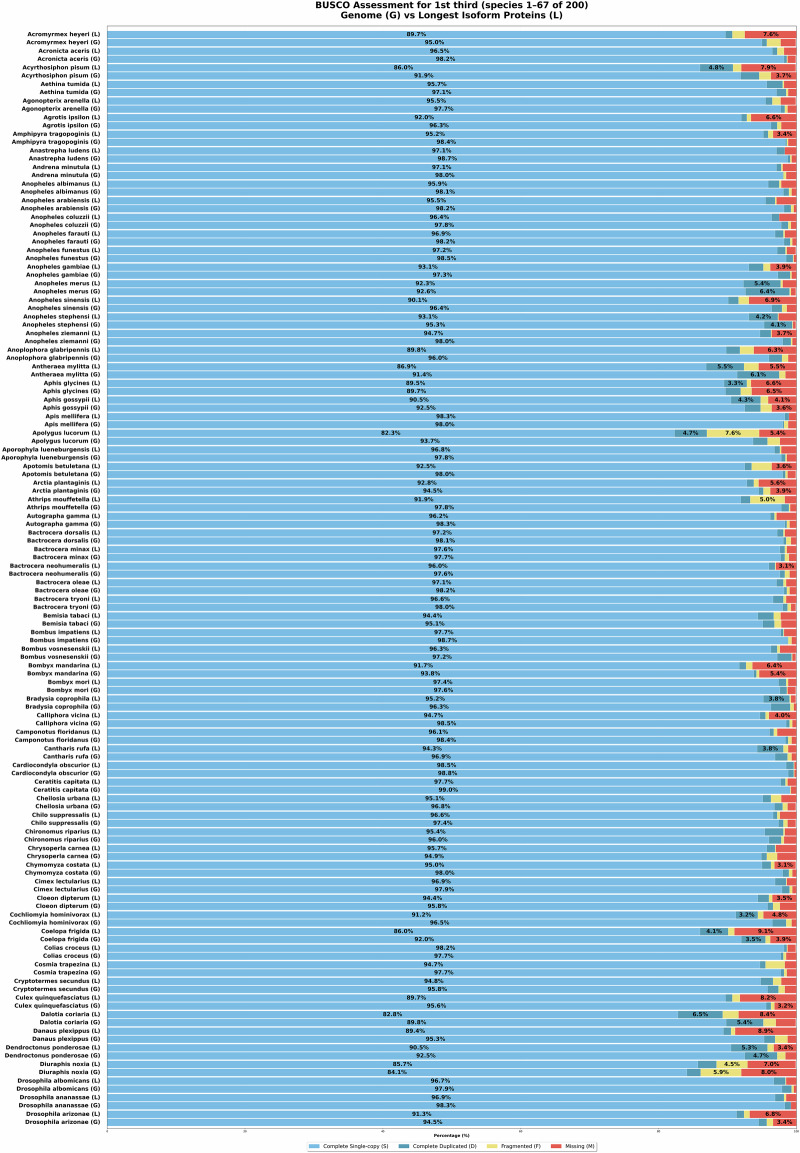
BUSCO scores of genome assemblies (G) and predicted genes by VARUS-BRAKER (B) (first third).

**Fig. 5:**
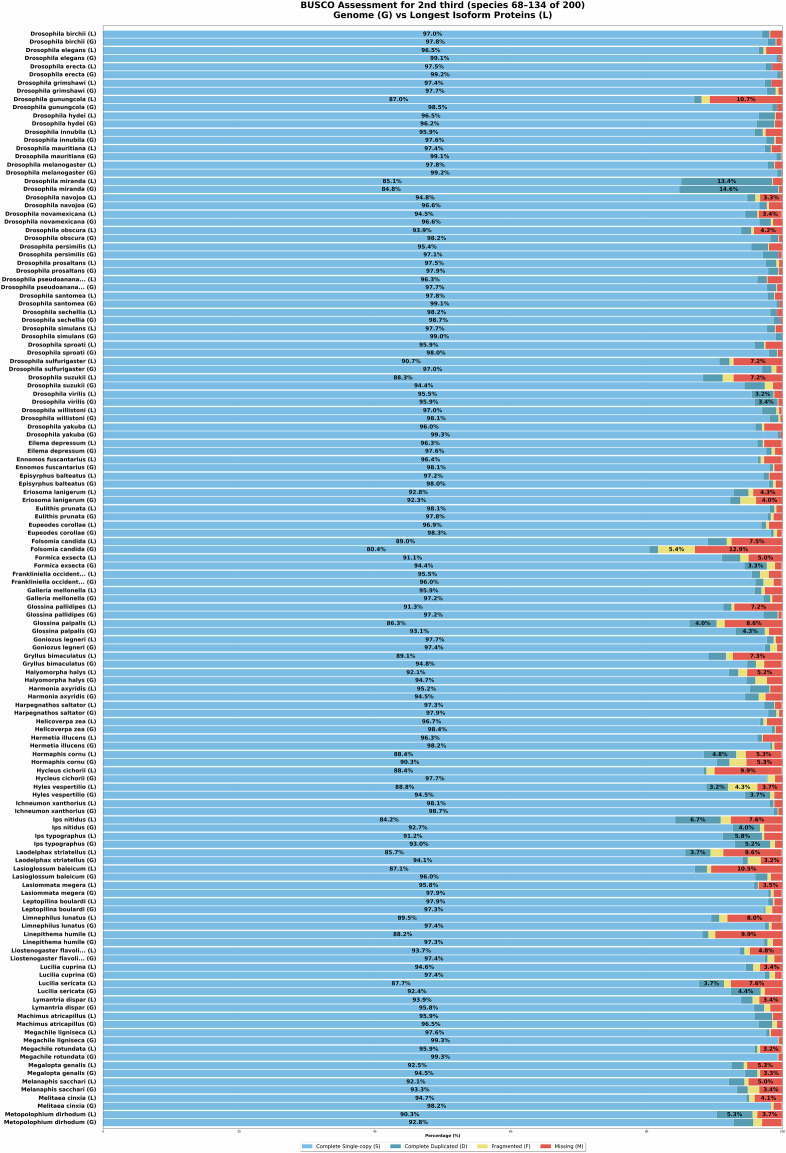
BUSCO scores of genome assemblies (G) and predicted genes by VARUS-BRAKER (B) (second third; continued).

**Fig. 6:**
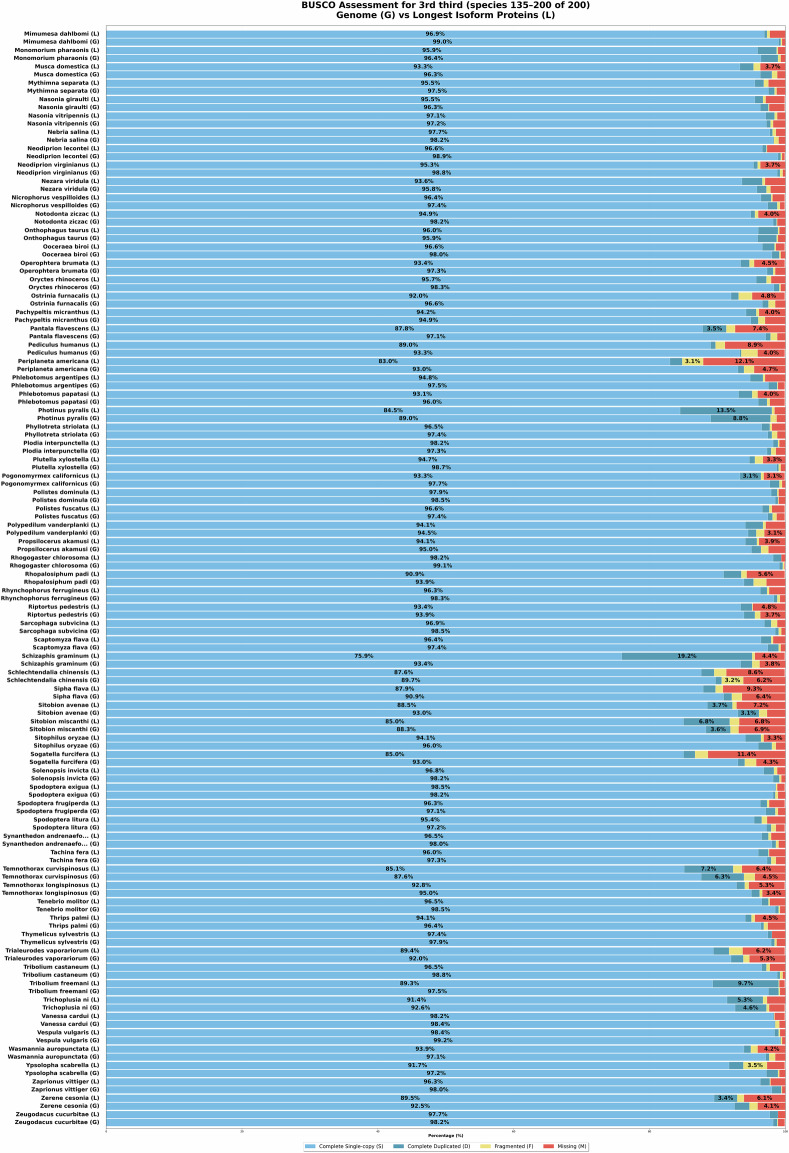
BUSCO scores of genome assemblies (G) and predicted genes by VARUS-BRAKER (B) (last third; continued).

With the above approach, initially, a total of 220 insect genome assemblies were retrieved from NCBI GenBank using the NCBI datasets^16^ tool. The data set includes genomic data from 77 families across 14 orders. Later, we had to exclude some species from the data set due to their unsatisfactory predicted protein completeness level. All the remaining 200 species obtain ≥85% completeness according to BUSCO, see Figs. 4–6. Nevertheless, we were able to maintain a balance between diversity and redundancy. Specifically, our taxon sampling includes the following species distributions: 40 from Lepidoptera, 34 from Hymenoptera, 24 from Hemiptera, and 20 from Coleoptera. A comprehensive list containing species, genus and accession numbers for the species’ genome assemblies, is provided in Supplementary Table S1.

### Structural genome annotation

All genome assemblies were processed with the VARUS-BRAKER workflow described above. In this use case with insect genomes, the workflow automatically retrieved the Arthropoda partition from OrthoDB v11^21^ from https://bioinf.uni-greifswald.de/bioinf/partitioned_odb11/Arthropoda.fa.gz. Since then, OrthoDB v12 has been integrated into the pipeline and is also available at https://bioinf.uni-greifswald.de/bioinf/partitioned_odb12.

The average running time of the workflow per species was approximately 20 hours, and 16 minutes, on an HPC node with Intel(R) Xeon(R) CPU E5-2650 v4 @2.20GHz using 36 CPUs, the runtime ranges from 5 h 9 min for *Goniozus legneri* to 78h 34 min for *Periplaneta americana*. This includes the time for automatic download of RNA-Seq data from NCBI, which varies due to the responsiveness of the SRA server.

**Fig. 7:**
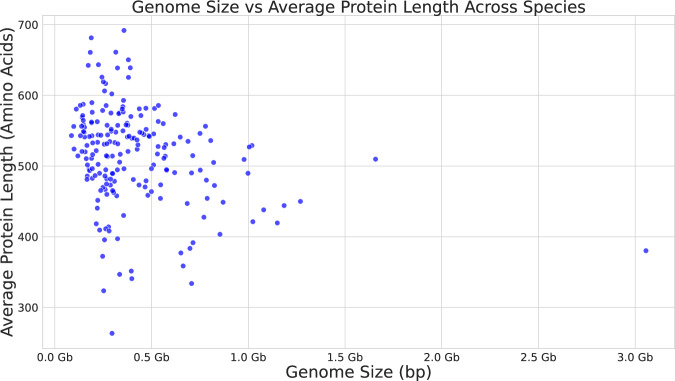
Distribution of genome and protein sizes.

**Fig. 8:**
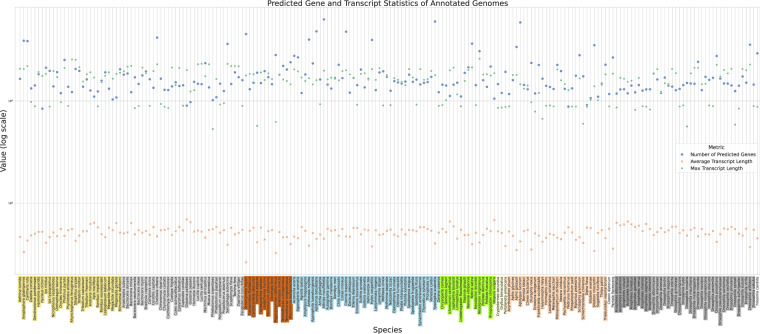
Protein length and number per species Coloured sectors indicate major insect clades: *Polyphaga* (yellow), flies *Diptera* (light gray), ants *Formicidae* (brown), moths *Ditrysia* (light blue), *Endopterygota* (green-yellow), *Polyneoptera* (light yellow), *Paraneoptera* (peach), *Palaleoptera* (silver) and *Drosophila* (gray/dark gray).

Figure 7 shows the distributions of genome sizes and predicted protein lengths. The average genome size is 413,245,217 bp, and the sizes range between a minimum of 86 Mb *Propsilocerus akamusi* and a maximum of 3 Gb for *Periplaneta americana*. Figure. 8 shows for each of the 200 species the number of predicted protein-coding genes and the average and maximum number of amino acids per protein sequence.

The 200 selected species are distributed across major insect clades as follows: **Endopterygota** (167 species), **Paraneoptera** (27), **Polyneoptera** (3), and **Palaeoptera** (3). We would like to point out that the purpose of the VARUS-BRAKER workflow is that users can annotate additional genomes or newer assemblies relatively easily.

### Orthology Analysis

The proteins predicted by BRAKER as part of the above workflow were grouped into orthogroups, representing sets of genes descended from a common ancestor. After annotating the 200 species listed in the Supplementary Table S1, we proceeded to find orthologous gene groups (orthogroups) in the whole set of 4,259,838 transcripts with OrthoFinder2 (v2.5.5)^30^, which performed an all-versus-all comparison of protein sequences. OrthoFinder2 was executed with the following command:


python orthofinder.py -f species_proteins_dir/ -M msa -A mafft -t 36 -a 36


Here, the -M msa flag specifies that multiple sequence alignment (MSA)-based orthogroup inference is used, and -A mafft indicates that MAFFT is the chosen alignment tool. The options -t 36 and -a 36 specify the number of threads allocated for the analysis. The Orthofinder2 pipeline also constructs a species tree with the STAG method (Species Tree inferred from All Orthogroups)^31^. The tree topologies of this tree and the tree constructed using RAxML-NG have a Robinson-Foulds distance^32^) of 4.9%, i.e. 95.1% of of all possible splits that are induced by the edges of either tree are shared by the other tree. Both trees are available in the deposited data.

Along the orthogroup analysis, protein multiple sequence alignments with MAFFT v7.505^33^ were constructed, the corresponding protein multiple sequence alignments files were used to construct a phylogenetic tree with RAxML Next Generation (RAxML-NG)^34^, using the LG+G8+F substitution model. This model consists of the fixed LG empirical amino acid substitution matrix, empirical amino acid frequencies inferred from the alignment (+F), and rate heterogeneity modeled with 8 discrete categories of a gamma distribution (+G8). Phylogenetic inference was performed with 200 bootstrap replicates. The resulting tree (200_insects_raxml.{pdf,nwk}) and the input alignment (SpeciesTreeAlignment.fa) are provided in the data deposited at figshare https://figshare.com/articles/dataset/Annotation_of_200_Insect_Genomes_with_BRAKER_for_Consistent_Comparisons_across_Species/28761460.

The tree was built with the following command:

raxml-ng –all –model LG+G8+F/WAG+G8+F –tree pars10

–bs-trees 200 –threads 36

–msa OrthoFinder2/Results/MultipleSequenceAlignments/SpeciesTreeAlignment.fa

Among the analyzed groups, all Endopterygota species were united into a hierarchical orthogroup, highlighting their shared evolutionary history.

The entire data processing workflow, including software versions, genome annotation, functional annotation and orthology analysis, was implemented using standardized tools and versions, as documented in the Methods section.

### Functional Annotation

#### FANTASIA

**Fig. 9:**
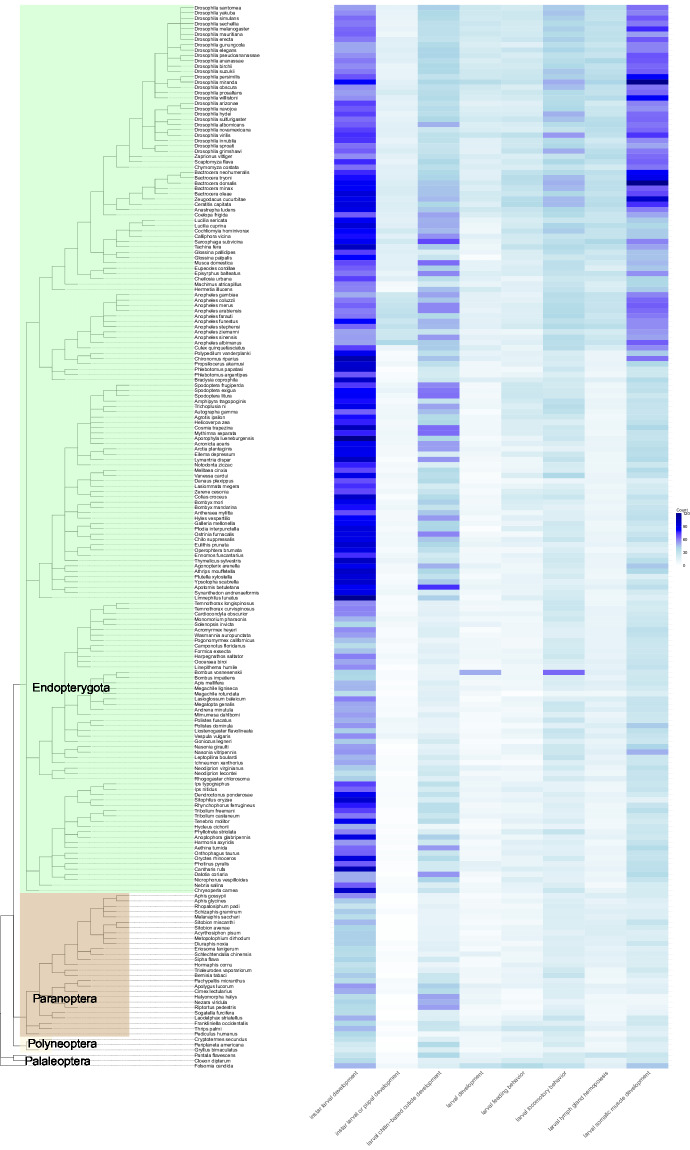
Visualization of an excerpt of the functional annotation. For selected biological processes as specified in gene ontology, the number of genes with this GO term annotation is shown.

Functional annotation was performed for all 200 species using the FANTASIA pipeline^28^, which assigns Gene Ontology (GO)^35^ IDs to transcripts. Multiple GO terms were assigned for some transcripts, reflecting their diverse functions. At the core of the FANTASIA pipeline is goPredSim^36^ with the protein language model ProtT5^37^, a similarity-based method for GO prediction. FANTASIA provides Gene Ontology terms for each protein sequence. While these identifiers are standardized and machine-readable, they are not immediately interpretable without mapping to their corresponding term names. To enhance interpretability, we applied an R-based post-processing step using the topGO package^38^ together with GO.db to map GO identifiers to their ontology categories: Biological Process (BP), Molecular Function (MF), and Cellular Component (CC). In this study, topGO was not used to perform Gene Ontology enrichment analyses; no statistical tests or enrichment algorithms were applied. Instead, it was used solely for reading gene-to-GO mappings and for organizing GO terms for annotation and visualization purposes. An example of applying this approach and analyzing the functional annotation data using GO terms is demonstrated in Fig. 9. For this figure, we used only isoforms labeled ‘larva’, or ‘pupa’ in the human-readable term.

#### InterProScan

We also performed a functional annotation with InterProScan v5.75-106.0^39^, a widely used domain-based approach, using the Pfam, PANTHER, and SUPERFAMILY databases.

#### Comparison between FANTASIA and InterProScan

We compared the coverage and agreement of annotations with GO terms of FANTASIA and InterProScan. Across the 200 insect proteomes, the above-described FANTASIA pipeline annotated 3,925,883 proteins with at least one GO term compared to 2,479,269 for InterProScan, corresponding to an approximately 1.58 times higher annotation rate. Among all 4,092,640 proteins with at least one GO prediction from either pipeline, 56.5% are annotated by both methods, 39.4% only by ProtT5 and 4.1% only by InterProScan; notably, 93.3% of InterProScan-annotated proteins are also annotated by FANTASIA, whereas 58.9% of FANTASIA-annotated proteins are annotated by InterProScan. Per species, median coverage is 95.9% for ProtT5 vs. 68.0% for InterProScan.

We quantify set-level overlap using the Jaccard index *J*_cov_(*A*, *B*) = |*A* ∩ *B*|/|*A* ∪ *B*|, where *A* and *B* are the sets of proteins with at least one GO term from either FANTASIA or InterProScan, respectively. The per-species median *J*_cov_ = 0.636 (IQR 0.562–0.672), and the pooled *J*_cov_ = 0.565.

Among proteins annotated by both methods, term-level agreement is high: 80.8% have a GO term shared between the two pipelines. This metric counts two GO terms as different that may be quite similar, such as a term like ‘DNA repair’ and ‘DNA damage response’, which is a parent of ‘DNA repair’ in the directed acyclic graph that GO defines. We therefore also computed a relaxed ontology-aware agreement between two term lists, in which two GO terms are considered agreeing if they share an ancestor that is at most 3 levels up from either term. For this, we did not allow a root term to be that ancestor. In this ontology-aware agreement metric, for 94.2% of genes the term lists of FANTASIA and InterProScan agreed in at least one term pair.

## Data Records

The whole dataset is publicly available for download on figshare^40^ https://figshare.com/articles/dataset/Annotation_of_200_Insect_Genomes_with_BRAKER_for_Consistent_Comparisons_across_Species/28761460, including annotations, protein sequences, GO terms and OrthoFinder2 analysis results. The dataset has a Creative Commons Attribution 4.0 International (CC BY 4.0) license. All species-specific data are stored in separate .tar.gz archives. Each such archive contains four files: (i) predicted protein sequences including all isoforms, (ii) predicted protein sequences containing only the longest isoform per gene, (iii) a structural annotation file, and (iv) a structural annotation file decorated with Gene Ontology terms in .gff3.gz format. For example, Drosophila_melanogaster.tar.gz contains the five files Drosophila_melanogaster{.faa,_longest.faa,.gtf,.gff3,_interproscan.tsv}.

Additionally, for user convenience, the structural annotation files decorated with Gene Ontology terms (.gff3.gz) have also been uploaded separately to allow direct automated access. This results in duplication, as the .gff3 files are available both individually and within the respective species archives. Furthermore, the results of OrthoFinder2 are provided in the archive OrthoFinder2_Results.tar.gz, and the results of tRNAscan-SE are provided in the archive tRNAscan_Results.tar.gz.

The genome sequences used as input for these annotations were obtained from NCBI. Details of the corresponding accession numbers, the specific BRAKER version used and whether the genomes had prior annotations are listed in Supplementary Table S1.

Regrettably, it was not possible to make the annotations produced in this study available via a third party annotation (TPA) submission to GenBank. The International Nucleotide Sequence Database Collaboration had announced in September 2024^41^ that third party annotations are not accepted anymore from January 2025.

### tRNA Prediction

As an additional layer of validation, we evaluated the non-coding RNA complement by predicting tRNAs across all 200 assemblies using tRNAscan-SE v2.0.12^42^. Key cross-species metrics include: a median of 489 confirmed tRNAs per genome (IQR 295-1,489), a median tRNA density of 3.63 per Mbp (IQR 2.40-6.76), and an estimated genomic occupancy of 0.0268% (IQR 0.0177-0.0501). The proportion of predicted pseudogenes is 19.75% (IQR 2.78-51.27), and 7.3% of non-pseudogene tRNAs contain introns (IQR 5.69-9.97). Selenocysteine tRNAs were detected in 46.0% of species and suppressor tRNAs in 42.5%. The most common isotypes by median share across species are Ser (~6.7%), Ala (~6.2%), Gly (~5.9%), Arg (~5.7%), and Leu (~5.6%).

## Technical Validation

### Gene Structure Accuracy

BRAKER3 was recently evaluated and compared against other pipelines in another study by comparing the predictions against reference annotations of 11 species^5^. The comparisons include the MAKER and Funannotate pipelines on 8 species, where BRAKER3 outperforms these two pipelines on all the usual accuracy metrics – sensitivity and precision on exon, gene and transcript level. For example, the gene-level sensitivity and precision on *D. melanogaster* are for BRAKER3 83.4%/90.6% vs 61.10%/52.8% for MAKER2 and 62.9%/63.0% for Funannotate. In doing these benchmarks, input protein evidence data from species closely related to the respective evaluation species were withheld. This was done to benchmark the application use case in which a new genome should be annotated. While Gabriel *et al*.^5^ manually selected complete RNA-Seq libraries, the VARUS-BRAKER workflow used here automatically *samples* from RNA-Seq data with VARUS^12^. In order to robustly compare the fully automatic VARUS-BRAKER workflow with BRAKER3 as benchmarked comparatively in^5^ we selected all 11 model organisms as benchmarks for VARUS-BRAKER as well. For each target species, we excluded all proteins from that same species in OrthoDB: *Danio rerio*, *Drosophila melanogaster*, *Gallus gallus*, *Mus musculus*, *Populus trichocarpa*, *Medicago truncatula*, *Parasteatoda tepidariorum*, *Arabidopsis thaliana*, *Solanum lycopersicum*, *Bombus terrestris* and *Caenorhabditis elegans*. In addition, we evaluated variations in batch size and the number of RNA-Seq datasets. Unlike the original BRAKER3 approach, in which SRA datasets were manually selected prior to testing, the RNA data provision process in this study was fully automated. We here demonstrate that the automatic selection of RNA-Seq datasets and their alignment to a reference genome does not compromise the quality of the results. The evaluated metrics were sensitivity, specificity, and the F1-score.

The results of the benchmarking are available in Table 1. Averaging over the 11 species, the accuracy of the VARUS-BRAKER workflow with automatic RNA-Seq sampling from among all libraries in SRA is very close to the accuracy of BRAKER3 when complete libraries were chosen manually. Gene- and transcript-level accuracy are slightly better in the automated workflow, and exon-level accuracy is slightly worse, all differences are at most 0.6 percent points. We therefore conclude that our fully automated VARUS-BRAKER workflow, which only requires the binomial names as manually prepared input, is on average as good as a semi-automatic approach, where RNA-Seq libraries are prepared manually.

**Table 1:** Gene Structure Accuracy (Sn = sensitivity, Pr = precision) of BRAKER3 in the original publication by^5^, and BRAKER3 in the VARUS-BRAKER workflow (arthropods in bold).

Species	Workflow	Gene		Transcript		Exon	
		Sn	Pr	Sn	Pr	Sn	Pr
***Drosophila melanogaster***	VARUS-BRAKER	87.85	90.31	61.19	83.11	84.16	95.43
	BRAKER3	85.67	89.89	58.66	83.69	82.88	95.75
***Bombus terrestris***	VARUS-BRAKER	68.83	67.02	50.25	62.01	73.70	90.93
	BRAKER3	72.51	66.34	52.53	60.08	78.56	90.10
***Parasteatoda tepidariorum***	VARUS-BRAKER	44.78	59.56	37.73	54.08	49.65	87.50
	BRAKER3	48.64	59.63	42.14	54.05	58.53	86.70
*Caenorhabditis elegans*	VARUS-BRAKER	71.54	85.34	54.21	76.23	78.67	94.84
	BRAKER3	68.22	85.54	51.86	77.64	75.25	95.45
*Arabidopsis thaliana*	VARUS-BRAKER	82.64	83.08	57.64	78.14	82.12	94.08
	BRAKER3	81.80	87.23	57.01	83.85	81.72	95.80
*Populus trichocarpa*	VARUS-BRAKER	78.33	88.62	63.40	81.52	85.59	94.82
	BRAKER3	77.75	88.31	63.08	82.00	85.08	94.99
*Medicago truncatula*	VARUS-BRAKER	50.56	72.95	50.56	65.56	77.71	88.46
	BRAKER3	49.62	73.14	49.62	66.57	76.89	88.98
*Danio rerio*	VARUS-BRAKER	56.84	71.11	35.30	68.06	65.85	92.73
	BRAKER3	56.91	66.60	35.83	58.23	65.15	90.79
*Gallus gallus*	VARUS-BRAKER	83.75	79.49	74.31	70.73	92.72	94.64
	BRAKER3	84.87	79.57	75.95	70.07	93.92	94.52
*Mus musculus*	VARUS-BRAKER	72.11	83.15	72.11	80.39	77.77	97.62
	BRAKER3	79.62	81.50	79.61	71.91	85.37	96.40
*Solanum lycopersicum*	VARUS-BRAKER	46.34	47.79	37.54	47.50	83.88	94.52
	BRAKER3	46.90	60.37	46.90	54.72	75.87	85.37
**Average**	VARUS-BRAKER	67.51	78.71	51.43	72.39	74.82	92.55
	BRAKER3	66.94	78.62	51.17	72.29	75.07	92.64

The following formulas were used to calculate the evaluation metrics:

- Let *TP*, *FP*, and *FN* denote the number of true positives, false positives, and false negatives, respectively.
- **Sensitivity (Sn)**: $$\frac{TP}{TP+FN}$$
- **Precision (Pr)**: $$\frac{TP}{TP+FP}$$
- **F1-score**: $$2\times \frac{\Pr \times {\rm{Sn}}}{\Pr +{\rm{Sn}}}$$

**Fig. 10:**
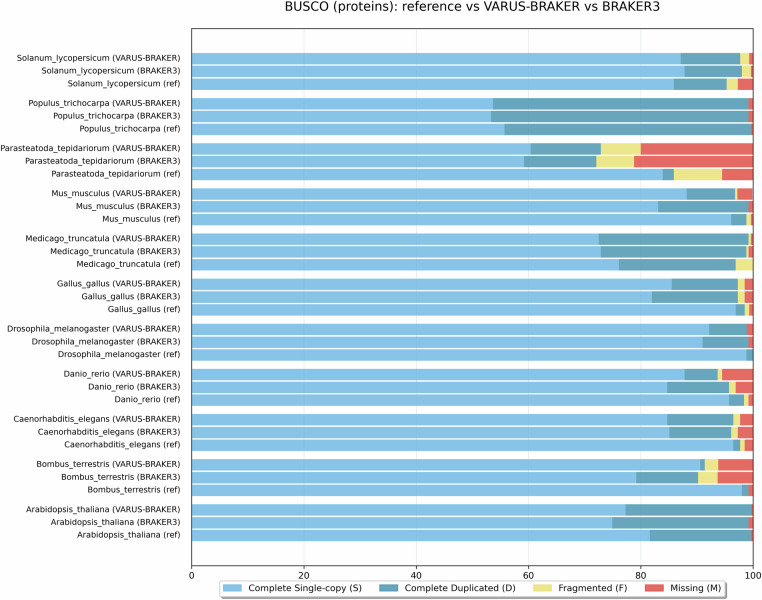
BUSCO assessment of protein annotations: reference, BRAKER3, and VARUS-BRAKER.

To complement these accuracy measures, we additionally assessed protein completeness using BUSCO and compared reference annotations, BRAKER3, and VARUS-BRAKER for the same benchmark species (Fig. 10). Across all species, BUSCO completeness values obtained with VARUS-BRAKER were highly similar to those obtained with BRAKER3. In several cases, VARUS-BRAKER showed a slightly higher fraction of complete single-copy BUSCOs. Reference annotations typically exhibited very high completeness but were dominated by duplicated BUSCOs, reflecting the presence of multiple annotated isoforms per gene. Overall, this analysis confirms that the fully automated VARUS-BRAKER workflow produces protein sets that are comparable in completeness and quality to semi-manual BRAKER3 annotations and consistent with existing reference resources.

### Proteome and Genome Completeness

To obtain one quality measure about proteome and genome completeness, we can use BUSCO^10^. For each species, the predicted proteomes and genomes were evaluated using a lineage-specific dataset, such as hymenoptera_odb11, lepidoptera_odb11, diptera_odb11 For the selected species, BUSCO showed an annotation completeness level of at least 85%. The BUSCO results are shown in Figs. 4–6.

In addition, we also performed BUSCO analyses directly on the genome assemblies used in this study. Across all 200 insect genomes analyzed, BUSCO revealed consistently high completeness scores: at least 90% completeness in 198/200 genomes (99.0%) and at least 95% completeness in 181/200 genomes (90.5%). The per-species values are provided in Figs. 4–6.

**Fig. 11:**
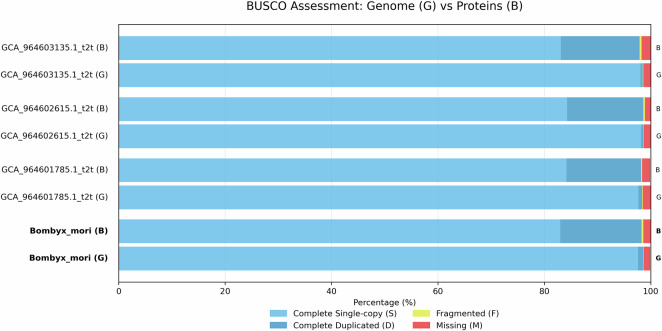
BUSCO scores of genome assemblies (G) and predicted genes by VARUS-BRAKER (B) for different silkworm *Bombyx mori* assemblies and predictions. The last genome and protein in this chart are the *Bombyx mori* assembly and annotation used for the downstream analysis (assembly accession GCF_014905235.1).

To further evaluate our workflow on state-of-the-art assemblies, we annotated three recently published telomere-to-telomere (T2T) genomes of the domesticated silkworm *Bombyx mori*^43^ using VARUS-BRAKER and assessed them with BUSCO. The T2T assemblies showed very high genome completeness (98.4 –98.6%) and similarly high completeness of protein annotations (97.9 –98.6%). These values were fully consistent with the BUSCO results obtained for the *Bombyx mori* assembly used in our dataset (genome completeness 98.6%, protein completeness 98.3%). The corresponding values are provided in Fig. 11. In this case of *Bombyx mori* assemblies, the less contiguous and complete assembly has a very similar BUSCO completeness, suggesting a small effect on protein-coding annotations.

### Overprediction or Contamination

**Fig. 12:**
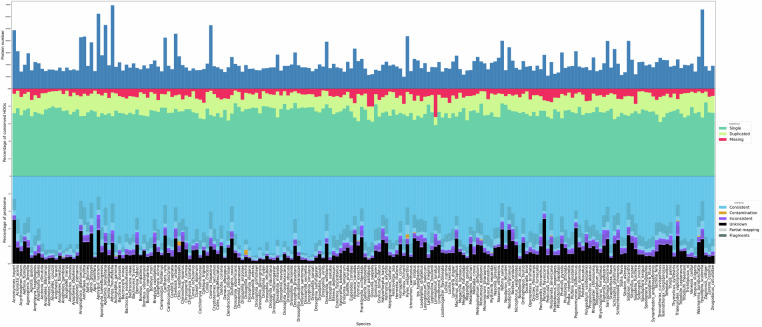
The top panel summarizes the overall proteome completeness by species, and the bottom panel shows the distribution of OMArk protein categories used to assess potential overestimation or contamination.

To obtain a second quality measure on the proteomes and to get additional information on overprediction or contamination, we ran OMArk. OMArk reports an average completeness level of 92% and shows low contamination levels (Fig. 12).

## Supplementary information

Species accession IDs, annotation status and corresponding reference.

## Data Availability

All annotation files generated in this study are available from Figshare^40^ at https://figshare.com/articles/dataset/Annotation_of_200_Insect_Genomes_with_BRAKER_for_Consistent_Comparisons_across_Species/28761460. The metadata.csv file deposited on Figshare provides NCBI/ENA accession numbers of the raw genome and transcriptome data used as input for the annotations. Readers can thus directly retrieve the original datasets from NCBI/ENA and apply the corresponding annotation files provided in this study.

## Code availability

The complete genome annotation workflow, including scripts and configurations for data processing, annotation, phylogenetic analysis with OrthoFinder, and Gene Ontology terms predictions with FANTASIA is available on GitHub at https://github.com/Gaius-Augustus/varus-braker/.

Further details on the workflow and additional custom scripts can be found in the repository documentation.
