# Supplementary material for: Annotation of 200 Insect Genomes with BRAKER for Consistent Comparisons across Species
Source: Sci Data. 2026 Feb 19;13:288. doi: 10.1038/s41597-026-06840-0 (PMC12923644; doi:10.1038/s41597-026-06840-0)
Supplement: Supplementary file 1 — Species accession IDs, annotation status and corresponding reference. [file 41597_2026_6840_MOESM1_ESM.pdf]

Annotation of 200 Insect Genomes with BRAKER for Consistent Comparisons across Species

Stepan Saenko[1], Katharina J. Hoff[1,2], Mario Stanke[1,2]

[1]Institute of Mathematics and Computer Science, University of Greifswald, Greifswald, 17489, Germany
[2]Center for Functional Genomics of Microbes, University of Greifswald, Greifswald, 17489, Germany

Supplementary Table: Annotation Summary of 200 Insect Genomes

Species	Accession ID	BRAKER 2 or 3	Annotation Status (GenBank)	Literature Reference
Acromyrmex heyeri	GCA_017607565.1	2	Annotated	[44]
Acronicta aceris	GCA_910591435.1	2	Not Annotated	[45]
Acyrthosiphon pisum	GCA_005508785.2	3	Annotated	[46]
Aethina tumida	GCA_024364675.1	3	Annotated	[47]
Agonopterix arenella	GCA_927399405.1	2	Not Annotated	[48]
Agrotis ipsilon	GCA_028554685.1	3	Not Annotated	[49]
Amphipyra tragopoginis	GCA_905220435.1	3	Not Annotated	[45]
Anastrepha ludens	GCA_028408465.1	3	Not Annotated	[47]
Andrena minutula	GCA_929113495.1	3	Not Annotated	[50]
Anopheles albimanus	GCA_013758885.1	3	Annotated	[51]
Anopheles arabiensis	GCA_016920715.1	3	Annotated	[52]
Anopheles coluzzii	GCA_943734685.1	3	Annotated	[45]
Anopheles farauti	GCA_000473445.2	3	Not Annotated	Direct Submission
Anopheles funestus	GCA_943734845.1	3	Annotated	[45]
Anopheles gambiae	GCA_000005575.1	3	Not Annotated	[53]
Anopheles merus	GCA_017562075.2	3	Annotated	Direct Submission
Anopheles sinensis	GCA_000441895.2	3	Annotated	[54]

Anopheles stephensi	GCA_013141755.1	3	Annotated	Direct Submission
Anopheles ziemanni	GCA_943734765.2	3	Not Annotated	[45]
Anoplophora glabripennis	GCA_000390285.2	3	Annotated	Direct Submission
Antheraea mylitta	GCA_014332785.1	2	Not Annotated	Direct Submission
Aphis glycines	GCA_009761285.1	3	Annotated	[55]
Aphis gossypii	GCA_020184175.2	2	Annotated	Direct Submission
Apis mellifera	GCA_003254395.2	3	Annotated	[56]
Apolygus lucorum	GCA_009739505.2	2	Annotated	[57]
Aporophyla lueneburgensis	GCA_932294355.1	3	Not Annotated	[45]
Apotomis betuletana	GCA_932273695.1	2	Not Annotated	[45]
Arctia plantaginis	GCA_902825455.1	3	Annotated	Direct Submission
Athrips mouffetella	GCA_947532105.1	2	Not Annotated	[45]
Autographa gamma	GCA_905146925.1	3	Not Annotated	[45]
Bactrocera dorsalis	GCA_023373825.1	3	Annotated	Direct Submission
Bactrocera minax	GCA_021498325.1	3	Not Annotated	Direct Submission
Bactrocera neohumeralis	GCA_024586455.2	3	Annotated	[58]
Bactrocera oleae	GCA_001188975.4	3	Annotated	Direct Submission
Bactrocera tryoni	GCA_016617805.2	3	Annotated	Direct Submission
Bemisia tabaci	GCA_001854935.1	3	Annotated	Direct Submission
Bombus impatiens	GCA_000188095.4	3	Annotated	[59]
Bombus vosnesenskii	GCA_011952255.1	3	Annotated	Direct Submission
Bombyx mandarina	GCA_003987935.1	3	Annotated	[60]
Bombyx mori	GCA_014905235.2	3	Annotated	Direct Submission
Bradysia coprophila	GCA_014529535.2	3	Annotated	Direct Submission
Calliphora vicina	GCA_958450345.1	3	Not Annotated	[45]
Camponotus floridanus	GCA_003227725.1	3	Annotated	[61]
Cantharis rufa	GCA_947369205.1	2	Not Annotated	[45]

Cardiocondyla obscurior	GCA_019399895.1	3	Not Annotated	Direct Submission
Ceratitis capitata	GCA_000347755.4	3	Annotated	Direct Submission
Cheilosia urbana	GCA_946477585.1	2	Not Annotated	[45]
Chilo suppressalis	GCA_902850365.2	3	Annotated	[62]
Chironomus riparius	GCA_917627325.4	3	Annotated	[62]
Chrysoperla carnea	GCA_905475395.1	3	Annotated	[45]
Chymomyza costata	GCA_018150985.1	3	Not Annotated	[63]
Cimex lectularius	GCA_000648675.3	3	Annotated	[64]
Cloeon dipterum	GCA_949628265.1	3	Annotated	Direct Submission
Cochliomyia hominivorax	GCA_004302925.2	3	Not Annotated	Direct Submission
Coelopa frigida	GCA_017309665.1	3	Not Annotated	Direct Submission
Colias croceus	GCA_905220415.1	3	Annotated	[45]
Cosmia trapezina	GCA_905163495.3	2	Not Annotated	[45]
Cryptotermes secundus	GCA_002891405.2	3	Annotated	[65]
Culex quinquefasciatus	GCA_015732765.1	3	Annotated	Direct Submission
Dalotia coriaria	GCA_025399875.2	3	Not Annotated	[66]
Danaus plexippus	GCA_009731565.1	3	Not Annotated	[67]
Dendroctonus ponderosae	GCA_020466585.2	3	Annotated	[68]
Diuraphis noxia	GCA_001186385.1	3	Annotated	[69]
Drosophila albomicans	GCA_009650485.2	2	Annotated	[70]
Drosophila ananassae	GCA_017639315.2	3	Annotated	[71]
Drosophila arizonae	GCA_001654025.1	3	Annotated	[72]
Drosophila birchii	GCA_008042755.1	3	Annotated	Direct Submission
Drosophila elegans	GCA_018152505.1	3	Annotated	[63]
Drosophila erecta	GCA_003286155.2	3	Annotated	Direct Submission
Drosophila grimshawi	GCA_018153295.1	3	Annotated	[63]
Drosophila gunungcola	GCA_025200985.1	3	Annotated	[73]
Drosophila hydei	GCA_003285905.2	3	Annotated	Direct Submission
Drosophila innubila	GCA_004354385.2	3	Annotated	[74]
Drosophila mauritiana	GCA_004382145.1	3	Annotated	Direct Submission

Drosophila melanogaster	GCA_000001215.4	3	Annotated	[75]
Drosophila miranda	GCA_003369915.2	3	Annotated	[76]
Drosophila navojoa	GCA_001654015.2	3	Annotated	[77]
Drosophila novamexicana	GCA_003285875.3	2	Annotated	Direct Submission
Drosophila obscura	GCA_018151105.1	3	Annotated	[63]
Drosophila persimilis	GCA_003286085.2	3	Annotated	Direct Submission
Drosophila prosaltans	GCA_018151275.1	2	Not Annotated	[63]
Drosophila pseudoananassae	GCA_018153035.1	3	Annotated	[78]
Drosophila santomea	GCA_016746245.2	3	Annotated	Direct Submission
Drosophila sechellia	GCA_004382195.2	3	Annotated	Direct Submission
Drosophila simulans	GCA_016746395.2	3	Annotated	Direct Submission
Drosophila sproati	GCA_018904355.1	3	Not Annotated	[63]
Drosophila sulfurigaster	GCA_023558435.1	3	Annotated	[70]
Drosophila suzukii	GCA_013340165.1	3	Annotated	Direct Submission
Drosophila virilis	GCA_003285735.2	3	Annotated	Direct Submission
Drosophila willistoni	GCA_018902025.2	2	Annotated	Direct Submission
Drosophila yakuba	GCA_016746365.2	3	Annotated	Direct Submission
Eilema depressum	GCA_914767945.1	3	Not Annotated	[45]
Ennomos fuscantarius	GCA_905220475.3	3	Not Annotated	[45]
Episyrphus balteatus	GCA_945859705.1	3	Not Annotated	[45]
Eriosoma lanigerum	GCA_013282895.1	3	Not Annotated	Direct Submission
Eulithis prunata	GCA_918843925.1	2	Not Annotated	[45]
Eupeodes corollae	GCA_945859685.1	3	Not Annotated	[45]
Folsomia candida	GCA_002217175.1	3	Annotated	[79]
Formica exsecta	GCA_003651465.1	3	Annotated	[80]
Frankliniella occidentalis	GCA_000697945.5	3	Annotated	[81]
Galleria mellonella	GCA_026898425.1	3	Annotated	Direct Submission

Glossina pallidipes	GCA_000688715.1	3	Not Annotated	Direct Submission
Glossina palpalis	GCA_000818775.1	3	Not Annotated	Direct Submission
Goniozus legneri	GCA_003055095.1	2	Not Annotated	Direct Submission
Gryllus bimaculatus	GCA_017312745.1	3	Not Annotated	[82]
Halyomorpha halys	GCA_000696795.3	3	Not Annotated	Direct Submission
Harmonia axyridis	GCA_914767665.1	3	Annotated	[45]
Harpegnathos saltator	GCA_003227715.2	3	Annotated	[61]
Helicoverpa zea	GCA_022581195.1	3	Annotated	[83]
Hermetia illucens	GCA_905115235.1	3	Annotated	Direct Submission
Hormaphis cornu	GCA_017140985.1	3	Not Annotated	[84]
Hycleus cichorii	GCA_013841215.1	3	Not Annotated	[85]
Hyles vespertilio	GCA_009982885.2	2	Not Annotated	[86]
Ichneumon xanthorius	GCA_917499995.1	3	Not Annotated	[45]
Ips nitidus	GCA_018691245.2	3	Not Annotated	[87]
Ips typographus	GCA_016097725.1	3	Not Annotated	[88]
Laodelphax striatellus	GCA_017141395.1	3	Annotated	[89]
Lasioglossum baleicum	GCA_022376115.1	2	Not Annotated	Direct Submission
Lasiommata megera	GCA_928268935.1	3	Not Annotated	[45]
Leptopilina boulardi	GCA_019393585.1	3	Not Annotated	Direct Submission
Limnephilus lunatus	GCA_917563855.2	3	Not Annotated	[45]
Linepithema humile	GCA_000217595.1	2	Annotated	[90]
Liostenogaster flavolineata	GCA_025433975.1	3	Not Annotated	Direct Submission
Lucilia cuprina	GCA_022045245.1	3	Annotated	Direct Submission
Lucilia sericata	GCA_015586225.1	3	Annotated	Direct Submission
Lymantria dispar	GCA_016802235.1	3	Not Annotated	[91]
Machimus atricapillus	GCA_933228815.1	3	Not Annotated	[45]
Megachile ligniseca	GCA_945859555.1	3	Not Annotated	[45]
Megachile rotundata	GCA_000220905.1	3	Annotated	Direct Submission
Megalopta genalis	GCA_011865705.1	3	Annotated	[92]

Melanaphis sacchari	GCA_002803265.2	3	Annotated	Direct Submission
Melitaea cinxia	GCA_905220565.1	3	Annotated	[93]
Metopolophium dirhodum	GCA_019925205.1	3	Not Annotated	Direct Submission
Mimumesa dahlbomi	GCA_917499265.3	2	Not Annotated	[45]
Monomorium pharaonis	GCA_013373865.2	3	Annotated	[94]
Musca domestica	GCA_000371365.1	3	Annotated	[95]
Mythimna separata	GCA_029852925.1	3	Not Annotated	[96]
Nasonia giraulti	GCA_016647725.1	3	Not Annotated	[97]
Nasonia vitripennis	GCA_009193385.2	3	Annotated	[98]
Nebria salina	GCA_944039245.1	2	Not Annotated	[45]
Neodiprion lecontei	GCA_021901455.1	3	Annotated	[64]
Neodiprion virginianus	GCA_021901495.1	2	Annotated	[64]
Nezara viridula	GCA_928085145.1	3	Annotated	[62]
Nicrophorus vespilloides	GCA_001412225.1	3	Annotated	[99]
Notodonta ziczac	GCA_918843915.1	3	Not Annotated	[45]
Onthophagus taurus	GCA_000648695.2	3	Annotated	Direct Submission
Ooceraea biroi	GCA_003672135.1	3	Annotated	[100]
Operophtera brumata	GCA_932527175.1	3	Annotated	[45]
Oryctes rhinoceros	GCA_020654165.1	3	Not Annotated	[101]
Ostrinia furnacalis	GCA_004193835.2	3	Not Annotated	Direct Submission
Pachypeltis micranthus	GCA_020466155.1	3	Not Annotated	[102]
Pantala flavescens	GCA_020796165.1	2	Not Annotated	Direct Submission
Pediculus humanus	GCA_000006295.1	3	Not Annotated	[103]
Periplaneta americana	GCA_025594305.2	3	Annotated	[104]
Phlebotomus argentipes	GCA_947086385.1	3	Not Annotated	Direct Submission
Phlebotomus papatasi	GCA_024763615.2	3	Not Annotated	Direct Submission
Photinus pyralis	GCA_008802855.1	3	Annotated	[105]
Phyllotreta striolata	GCA_918026865.1	3	Annotated	[62]
Plodia interpunctella	GCA_027563975.1	3	Annotated	[47]
Plutella xylostella	GCA_932276165.1	3	Annotated	[45]
Pogonomyrmex californicus	GCA_024349325.1	3	Not Annotated	[106]

Polistes dominula	GCA_001465965.1	2	Annotated	[107]
Polistes fuscatus	GCA_010416935.1	3	Annotated	[108]
Polypedilum vanderplanki	GCA_018290095.1	3	Annotated	Direct Submission
Propsilocerus akamusi	GCA_018397935.1	3	Not Annotated	[109]
Rhogogaster chlorosoma	GCA_944452935.1	3	Not Annotated	[45]
Rhopalosiphum padi	GCA_020882245.1	3	Not Annotated	Direct Submission
Rhynchophorus ferrugineus	GCA_030347505.1	3	Annotated	[110]
Riptortus pedestris	GCA_019009955.1	3	Not Annotated	[111]
Sarcophaga subvicina	GCA_936449025.2	2	Not Annotated	[45]
Scaptomyza flava	GCA_030179655.1	3	Not Annotated	[112]
Schizaphis graminum	GCA_020882235.1	3	Not Annotated	Direct Submission
Schlechtendalia chinensis	GCA_019022885.1	3	Not Annotated	[70]
Sipha flava	GCA_003268045.1	3	Annotated	Direct Submission
Sitobion avenae	GCA_019425605.1	3	Not Annotated	Direct Submission
Sitobion miscanthi	GCA_008086715.1	2	Not Annotated	[113]
Sitophilus oryzae	GCA_002938485.2	3	Annotated	[114]
Sogatella furcifera	GCA_017141385.1	3	Not Annotated	Direct Submission
Solenopsis invicta	GCA_016802725.1	3	Annotated	Direct Submission
Spodoptera exigua	GCA_902829305.4	3	Annotated	[62]
Spodoptera frugiperda	GCA_023101765.3	3	Annotated	[115]
Spodoptera litura	GCA_002706865.3	3	Annotated	[116]
Synanthedon andrenaeformis	GCA_936446665.2	3	Not Annotated	[45]
Tachina fera	GCA_905220375.1	3	Not Annotated	[45]
Temnothorax curvispinosus	GCA_003070985.1	3	Annotated	Direct Submission
Temnothorax longispinosus	GCA_030848805.1	3	Annotated	[117]
Tenebrio molitor	GCA_907166875.3	3	Annotated	[118]
Thrips palmi	GCA_012932325.1	3	Annotated	[119]
Thymelicus sylvestris	GCA_911387775.1	3	Not Annotated	[45]
Trialeurodes vaporariorum	GCA_011764245.1	2	Not Annotated	Direct Submission
Tribolium castaneum	GCA_000002335.3	3	Annotated	[120]

Tribolium freemani	GCA_939628115.1	3	Not Annotated	Direct Submission
Trichoplusia ni	GCA_003590095.1	3	Annotated	[121]
Vanessa cardui	GCA_905220365.2	3	Annotated	[45]
Vespula vulgaris	GCA_905475345.1	3	Annotated	[45]
Wasmannia auropunctata	GCA_000956235.1	2	Annotated	Direct Submission
Ypsolopha scabrella	GCA_910592155.1	2	Not Annotated	[45]
Zaprionus vittiger	GCA_018904025.1	2	Not Annotated	[63]
Zerene cesonia	GCA_012273895.2	3	Annotated	[122]
Zeugodacus cucurbitae	GCA_028554725.2	3	Not Annotated	Direct Submission

Supplementary Table S1: Species accession IDs, annotation status and corresponding reference

References

44. Schrader, L. *et al.* Relaxed selection underlies genome erosion in socially parasitic ant species. *Nature Communications* **12.** ISSN: 2041-1723. http://dx.doi.org/10.1038/s41467-021-23178-w (May 2021).

45. Darwin Tree of Life Consortium. *Darwin Tree of Life Project* https://www.darwintreeoflife.org/. Accessed 2025-08-29. Wellcome Sanger Institute et al., 2025.

46. Li, Y., Park, H., Smith, T. E. & Moran, N. A. Gene Family Evolution in the Pea Aphid Based on Chromosome-Level Genome Assembly. *Molecular Biology and Evolution* **36** (ed Singh, N.) 2143–2156. ISSN: 1537-1719. http://dx.doi.org/10.1093/molbev/msz138 (June 2019).

47. USDA ARS. *Ag100Pest Initiative* https://www.ars.usda.gov/ag100pest/. Accessed 2025-08-29. United States Department of Agriculture, Agricultural Research Service, 2025.

48. Boyes, D., Crowley, L. & Holland, P. W. The genome sequence of the sycamore, Acronicta aceris (Linnaeus, 1758). *Wellcome Open Research* **6,** 326. ISSN: 2398-502X. http://dx.doi.org/10.12688/wellcomeopenres.17354.1 (Nov. 2021).

49. Jin, M. *et al.* Chromosome-level genome of black cutworm provides novel insights into polyphagy and seasonal migration in insects. *BMC Biology* **21.** ISSN: 1741-7007. http://dx.doi.org/10.1186/s12915-022-01504-y (Jan. 2023).

50. Falk, S. & Blomfield-Smith, H. The genome sequence of the common mini-mining bee Andrena minutula (Kirby, 1802). *Wellcome Open Research* **7,** 300. ISSN: 2398-502X. http://dx.doi.org/10.12688/wellcomeopenres.18611.1 (Dec. 2022).

51. Compton, A. *et al.* The Beginning of the End: A Chromosomal Assembly of the New World Malaria Mosquito Ends with a Novel Telomere. *G3 Genes—Genomes—Genetics* **10,** 3811–3819. ISSN: 2160-1836. http://dx.doi.org/10.1534/g3.120.401654 (Oct. 2020).

52. Zamyatin, A. *et al.* Chromosome-level genome assemblies of the malaria vectors Anopheles coluzzii and Anopheles arabiensis. *GigaScience* **10.** ISSN: 2047-217X. http://dx.doi.org/10.1093/gigascience/giab017 (Mar. 2021).

53. Holt, R. A. *et al.* The Genome Sequence of the Malaria Mosquito Anopheles gambiae. *Science* **298,** 129–149. ISSN: 1095-9203. http://dx.doi.org/10.1126/science.1076181 (Oct. 2002).

54. Zhou, D. *et al.* Genome sequence of Anopheles sinensis provides insight into genetics basis of mosquito competence for malaria parasites. *BMC Genomics* **15.** ISSN: 1471-2164. http://dx.doi.org/10.1186/1471-2164-15-42 (Jan. 2014).

55. Giordano, R. *et al.* Soybean aphid biotype 1 genome: Insights into the invasive biology and adaptive evolution of a major agricultural pest. *Insect Biochemistry and Molecular Biology* **120,** 103334. ISSN: 0965-1748. http://dx.doi.org/10.1016/j.ibmb.2020.103334 (May 2020).

56. Wallberg, A. *et al.* A hybrid de novo genome assembly of the honeybee, Apis mellifera, with chromosome-length scaffolds. *BMC Genomics* **20.** ISSN: 1471-2164. http://dx.doi.org/10.1186/s12864-019-5642-0 (Apr. 2019).

57. Liu, Y. *et al.* Apolygus lucorum genome provides insights into omnivorousness and mesophyll feeding. *Molecular Ecology Resources* **21,** 287–300. ISSN: 1755-0998. http://dx.doi.org/10.1111/1755-0998.13253 (Oct. 2020).

58. Castro-Vargas, C. *et al.* Genetic variation for rectal gland volatiles among recently collected isofemale lines and a domesticated strain of Queensland fruit fly, Bactrocera tryoni (Diptera: Tephritidae). *PLOS ONE* **18** (ed Nehring, V.) e0285099. ISSN: 1932-6203. http://dx.doi.org/10.1371/journal.pone.0285099 (Apr. 2023).

59. Sadd, B. M. *et al.* The genomes of two key bumblebee species with primitive eusocial organization. *Genome Biology* **16.** ISSN: 1465-6906. http://dx.doi.org/10.1186/s13059-015-0623-3 (Apr. 2015).

60. Xiang, H. *et al.* The evolutionary road from wild moth to domestic silkworm. *Nature Ecology & Evolution* **2,** 1268–1279. ISSN: 2397-334X. http://dx.doi.org/10.1038/s41559-018-0593-4 (July 2018).

61. Shields, E. J., Sheng, L., Weiner, A. K., Garcia, B. A. & Bonasio, R. High-Quality Genome Assemblies Reveal Long Non-coding RNAs Expressed in Ant Brains. *Cell Reports* **23,** 3078–3090. ISSN: 2211-1247. http://dx.doi.org/10.1016/j.celrep.2018.05.014 (June 2018).

62. Pest Genomics Initiative. *Pest Genomics Initiative* https://www.pestgenomics.org/. Accessed 2025-08-29. Consortium, 2025.

63. Kim, B. Y. *et al.* Highly contiguous assemblies of 101 drosophilid genomes. *eLife* **10.** ISSN: 2050-084X. http://dx.doi.org/10.7554/eLife.66405 (July 2021).

64. i5K Consortium. *The i5K Initiative: 5,000 Arthropod Genomes* https://i5k.github.io/. Accessed 2025-08-29. USDA ARS; Baylor College of Medicine; partners, 2025.

65. Harrison, M. C. *et al.* Hemimetabolous genomes reveal molecular basis of termite eusociality. *Nature Ecology & Evolution* **2,** 557–566. ISSN: 2397-334X. http://dx.doi.org/10.1038/s41559-017-0459-1 (Feb. 2018).

66. Kitchen, S. A. *et al.* The genomic and cellular basis of biosynthetic innovation in rove beetles. http://dx.doi.org/10.1101/2023.05.29.542378 (May 2023).

67. Gu, L. *et al.* Dichotomy of Dosage Compensation along the Neo Z Chromosome of the Monarch Butterfly. *Current Biology* **29,** 4071–4077.e3. ISSN: 0960-9822. http://dx.doi.org/10.1016/j.cub.2019.09.056 (Dec. 2019).

68. Keeling, C. I. *et al.* Chromosome-level genome assembly reveals genomic architecture of northern range expansion in the mountain pine beetle, Dendroctonus ponderosae Hopkins (Coleoptera: Curculionidae). *Molecular Ecology Resources* **22,** 1149–1167. ISSN: 1755-0998. http://dx.doi.org/10.1111/1755-0998.13528 (Oct. 2021).

69. Nicholson, S. J. *et al.* The genome of Diuraphis noxia, a global aphid pest of small grains. *BMC Genomics* **16.** ISSN: 1471-2164. http://dx.doi.org/10.1186/s12864-015-1525-1 (June 2015).

70. Wei, H. *et al.* Chromosome-level genome assembly for the horned-gall aphid provides insights into interactions between gall-making insect and its host plant. *Ecology and Evolution* **12.** ISSN: 2045-7758. http://dx.doi.org/10.1002/ece3.8815 (Apr. 2022).

71. Tvedte, E. S. *et al.* Comparison of long-read sequencing technologies in interrogating bacteria and fly genomes. *G3 Genes—Genomes—Genetics* **11** (ed Baltrus, D.) ISSN: 2160-1836. http://dx.doi.org/10.1093/g3journal/jkab083 (Apr. 2021).

72. Sanchez-Flores, A. *et al.* Genome Evolution in Three Species of CactophilicDrosophila. *G3 Genes—Genomes—Genetics* **6,** 3097–3105. ISSN: 2160-1836. http://dx.doi.org/10.1534/g3.116.033779 (Oct. 2016).

73. Negi, A., Liao, B.-Y. & Yeh, S.-D. Long-read-based Genome Assembly ofDrosophila gunungcolaReveals Fewer Chemosensory Genes in Flower-breeding Species. *Genome Biology and Evolution* **15** (ed Wheat, C.) ISSN: 1759-6653. http://dx.doi.org/10.1093/gbe/evad048 (Mar. 2023).

74. Hill, T., Koseva, B. S. & Unckless, R. L. The Genome of Drosophila innubila Reveals Lineage-Specific Patterns of Selection in Immune Genes. *Molecular Biology and Evolution* **36** (ed Singh, N.) 1405–1417. ISSN: 1537-1719. http://dx.doi.org/10.1093/molbev/msz059 (Mar. 2019).

75. Adams, M. D. *et al.* The Genome Sequence of Drosophila melanogaster. *Science* **287,** 2185–2195. ISSN: 1095-9203. http://dx.doi.org/10.1126/science.287.5461.2185 (Mar. 2000).

76. Mahajan, S., Wei, K. H.-C., Nalley, M. J., Gibilisco, L. & Bachtrog, D. De novo assembly of a young Drosophila Y chromosome using single-molecule sequencing and chromatin conformation capture. *PLOS Biology* **16** (ed Tyler-Smith, C.) e2006348. ISSN: 1545-7885. http://dx.doi.org/10.1371/journal.pbio.2006348 (July 2018).

77. Vanderlinde, T., Dupim, E. G., Nazario-Yepiz, N. O. & Carvalho, A. B. An Improved Genome Assembly for Drosophila navojoa, the Basal Species in the mojavensis Cluster. *Journal of Heredity* **110,** 118–123. ISSN: 1465-7333. http://dx.doi.org/10.1093/jhered/esy059 (Nov. 2018).

78. Gebert, D. *et al.* Analysis of 30 chromosome-levelDrosophilagenome assemblies reveals dynamic evolution of centromeric satellite repeats. http://dx.doi.org/10.1101/2024.06.17.599346 (June 2024).

79. Faddeeva-Vakhrusheva, A. *et al.* Coping with living in the soil: the genome of the parthenogenetic springtail Folsomia candida. *BMC Genomics* **18.** ISSN: 1471-2164. http://dx.doi.org/10.1186/s12864-017-3852-x (June 2017).

80. Dhaygude, K., Nair, A., Johansson, H., Wurm, Y. & Sundström, L. The first draft genomes of the ant Formica exsecta, and its Wolbachia endosymbiont reveal extensive gene transfer from endosymbiont to host. *BMC Genomics* **20.** ISSN: 1471-2164. http://dx.doi.org/10.1186/s12864-019-5665-6 (Apr. 2019).

81. Rotenberg, D. *et al.* Genome-enabled insights into the biology of thrips as crop pests. *BMC Biology* **18.** ISSN: 1741-7007. http://dx.doi.org/10.1186/s12915-020-00862-9 (Oct. 2020).

82. Ylla, G. *et al.* Insights into the genomic evolution of insects from cricket genomes. *Communications Biology* **4.** ISSN: 2399-3642. http://dx.doi.org/10.1038/s42003-021-02197-9 (June 2021).

83. Stahlke, A. R. *et al.* A Chromosome-Scale Genome Assembly of a Helicoverpa zea Strain Resistant to Bacillus thuringiensis Cry1Ac Insecticidal Protein. *Genome Biology and Evolution* **15** (ed Wheat, C.) ISSN: 1759-6653. http://dx.doi.org/10.1093/gbe/evac131 (Aug. 2022).

84. Korgaonkar, A. *et al.* A novel family of secreted insect proteins linked to plant gall development. *Current Biology* **31,** 1836–1849.e12. ISSN: 0960-9822. http://dx.doi.org/10.1016/j.cub.2021.01.104 (May 2021).

85. Wu, Y.-M., Li, J. & Chen, X.-S. Draft genomes of two blister beetles Hycleus cichorii and Hycleus phaleratus. *GigaScience* **7.** ISSN: 2047-217X. http://dx.doi.org/10.1093/gigascience/giy006 (Feb. 2018).

86. Pippel, M. *et al.* A highly contiguous genome assembly of the bat hawkmoth Hyles vespertilio (Lepidoptera: Sphingidae). *GigaScience* **9.** ISSN: 2047-217X. http://dx.doi.org/10.1093/gigascience/giaa001 (Jan. 2020).

87. Wang, Z. *et al.* Genome and transcriptome of Ips nitidus provide insights into high-altitude hypoxia adaptation and symbiosis. *iScience* **26,** 107793. ISSN: 2589-0042. http://dx.doi.org/10.1016/j.isci.2023.107793 (Oct. 2023).

88. Powell, D. *et al.* A highly contiguous genome assembly of a major forest pest, the Eurasian spruce bark beetleIps typographus. http://dx.doi.org/10.1101/2020.11.28.401976 (Nov. 2020).

89. Hu, Q.-L. *et al.* Chromosome-level Assembly, Dosage Compensation and Sex-biased Gene Expression in the Small Brown Planthopper,Laodelphax striatellus. *Genome Biology and Evolution* **14** (ed Mank, J.) ISSN: 1759-6653. http://dx.doi.org/10.1093/gbe/evac160 (Nov. 2022).

90. Smith, C. D. *et al.* Draft genome of the globally widespread and invasive Argentine ant (Linepithema humile). *Proceedings of the National Academy of Sciences* **108,** 5673–5678. ISSN: 1091-6490. http://dx.doi.org/10.1073/pnas.1008617108 (Jan. 2011).

91. Sparks, M. E. *et al.* Sequencing, assembly and annotation of the whole-insect genome of Lymantria dispar dispar, the European gypsy moth. *G3 Genes—Genomes—Genetics* **11** (ed Oliver, B.) ISSN: 2160-1836. http://dx.doi.org/10.1093/g3journal/jkab150 (Apr. 2021).

92. Kapheim, K. M. *et al.* Developmental plasticity shapes social traits and selection in a facultatively eusocial bee. *Proceedings of the National Academy of Sciences* **117,** 13615–13625. ISSN: 1091-6490. http://dx.doi.org/10.1073/pnas.2000344117 (May 2020).

93. Ahola, V. *et al.* The Glanville fritillary genome retains an ancient karyotype and reveals selective chromosomal fusions in Lepidoptera. *Nature Communications* **5.** ISSN: 2041-1723. http://dx.doi.org/10.1038/ncomms5737 (Sept. 2014).

94. Gao, Q. *et al.* High-quality chromosome-level genome assembly and full-length transcriptome analysis of the pharaoh ant Monomorium pharaonis. *GigaScience* **9.** ISSN: 2047-217X. http://dx.doi.org/10.1093/gigascience/giaa143 (Dec. 2020).

95. Scott, J. G. *et al.* Genome of the house fly, Musca domestica L., a global vector of diseases with adaptations to a septic environment. *Genome Biology* **15.** ISSN: 1474-760X. http://dx.doi.org/10.1186/s13059-014-0466-3 (Oct. 2014).

96. Zhao, H. *et al.* Chromosome-level genomes of two armyworms, Mythimna separata and Mythimna loreyi, provide insights into the biosynthesis and reception of sex pheromones. *Molecular Ecology Resources* **23,** 1423–1441. ISSN: 1755-0998. http://dx.doi.org/10.1111/1755-0998.13809 (May 2023).

97. Wang, X. *et al.* Genome Report: Whole Genome Sequence and Annotation of the Parasitoid Jewel Wasp Nasonia giraulti Laboratory Strain RV2X[u]. *G3 Genes—Genomes—Genetics* **10,** 2565–2572. ISSN: 2160-1836. http://dx.doi.org/10.1534/g3.120.401200 (Aug. 2020).

98. Dalla Benetta, E. *et al.* Genome elimination mediated by gene expression from a selfish chromosome. *Science Advances* **6.** ISSN: 2375-2548. http://dx.doi.org/10.1126/sciadv.aaz9808 (Apr. 2020).

99. Cunningham, C. B. *et al.* The Genome and Methylome of a Beetle with Complex Social Behavior,Nicrophorus vespilloides(Coleoptera: Silphidae). *Genome Biology and Evolution* **7,** 3383–3396. ISSN: 1759-6653. http://dx.doi.org/10.1093/gbe/evv194 (Oct. 2015).

100. McKenzie, S. K. & Kronauer, D. J. The genomic architecture and molecular evolution of ant odorant receptors. *Genome Research* **28,** 1757–1765. ISSN: 1549-5469. http://dx.doi.org/10.1101/gr.237123.118 (Sept. 2018).

101. Filipović, I. *et al.* A high-quality de novo genome assembly based on nanopore sequencing of a wild-caught coconut rhinoceros beetle (Oryctes rhinoceros). *BMC Genomics* **23.** ISSN: 1471-2164. http://dx.doi.org/10.1186/s12864-022-08628-z (June 2022).

102. Wang, X., Zhao, N., Cai, L., Liu, N. & Yang, B. Genome sequencing of Pachypeltis micranthus Mu et Liu (Hemiptera: Miridae), a potential biological control agent for Mikania micrantha. http://dx.doi.org/10.22541/au.170668451.17862296/v1 (Jan. 2024).

103. Kirkness, E. F. *et al.* Genome sequences of the human body louse and its primary endosymbiont provide insights into the permanent parasitic lifestyle. *Proceedings of the National Academy of Sciences* **107,** 12168–12173. ISSN: 1091-6490. http://dx.doi.org/10.1073/pnas.1003379107 (June 2010).

104. Wang, L. *et al.* Genome assembly and annotation of Periplaneta americana reveal a comprehensive cockroach allergen profile. *Allergy* **78,** 1088–1103. ISSN: 1398-9995. http://dx.doi.org/10.1111/all.15531 (Oct. 2022).

105. Fallon, T. R. *et al.* Firefly genomes illuminate parallel origins of bioluminescence in beetles. *eLife* **7.** ISSN: 2050-084X. http://dx.doi.org/10.7554/eLife.36495 (Oct. 2018).

106. Errbii, M. *et al.* Evolutionary genomics of socially polymorphic populations ofPogonomyrmex californicus. http://dx.doi.org/10.1101/2021.03.21.436260 (Mar. 2021).

107. Standage, D. S. *et al.* Genome, transcriptome and methylome sequencing of a primitively eusocial wasp reveal a greatly reduced DNA methylation system in a social insect. *Molecular Ecology* **25,** 1769–1784. ISSN: 1365-294X. http://dx.doi.org/10.1111/mec.13578 (Mar. 2016).

108. Miller, S. E. *et al.* Evolutionary dynamics of recent selection on cognitive abilities. *Proceedings of the National Academy of Sciences* **117,** 3045–3052. ISSN: 1091-6490. http://dx.doi.org/10.1073/pnas.1918592117 (Jan. 2020).

109. Sun, X. *et al.* A chromosome level genome assembly of Propsilocerus akamusi to understand its response to heavy metal exposure. *Molecular Ecology Resources* **21,** 1996–2012. ISSN: 1755-0998. http://dx.doi.org/10.1111/1755-0998.13377 (Mar. 2021).

110. Hazzouri, K. M. *et al.* The genome of pest Rhynchophorus ferrugineus reveals gene families important at the plant-beetle interface. *Communications Biology* **3.** ISSN: 2399-3642. http://dx.doi.org/10.1038/s42003-020-1060-8 (June 2020).

111. Huang, H. *et al.* Chromosome-level genome assembly of the bean bug Riptortus pedestris. *Molecular Ecology Resources* **21,** 2423–2436. ISSN: 1755-0998. http://dx.doi.org/10.1111/1755-0998.13434 (June 2021).

112. Pelaez, J. N. *et al.* Evolution of chemosensory and detoxification gene families across herbivorous Drosophilidae. http://dx.doi.org/10.1101/2023.03.16.532987 (Mar. 2023).

113. Jiang, X. *et al.* A chromosome-level draft genome of the grain aphid Sitobion miscanthi. *GigaScience* **8.** ISSN: 2047-217X. http://dx.doi.org/10.1093/gigascience/giz101 (Aug. 2019).

114. Parisot, N. *et al.* The transposable element-rich genome of the cereal pest Sitophilus oryzae. *BMC Biology* **19.** ISSN: 1741-7007. http://dx.doi.org/10.1186/s12915-021-01158-2 (Nov. 2021).

115. Rane, R. *et al.* *Chromosomal genome assembly for Spodoptera frugiperda (fall armyworm)* Data collection. Version 1 (2022). https://hdl.handle.net/102.100.100/489008?index=1.

116. Cheng, T. *et al.* Genomic adaptation to polyphagy and insecticides in a major East Asian noctuid pest. *Nature Ecology & Evolution* **1,** 1747–1756. ISSN: 2397-334X. http://dx.doi.org/10.1038/s41559-017-0314-4 (Sept. 2017).

117. Jongepier, E. *et al.* Convergent Loss of Chemoreceptors across Independent Origins of Slave-Making in Ants. *Molecular Biology and Evolution* **39** (ed Wittkopp, P.) ISSN: 1537-1719. http://dx.doi.org/10.1093/molbev/msab305 (Oct. 2021).

118. Genoscope – CEA. *Genoscope (CEA) Genome Resources* https://www.genoscope.cns.fr/. Accessed 2025-08-29. Commissariat à l'Énergie Atomique et aux Énergies Alternatives, 2025.

119. Guo, S. *et al.* Chromosome-level assembly of the melon thrips genome yields insights into evolution of a sap-sucking lifestyle and pesticide resistance. *Molecular Ecology Resources* **20,** 1110–1125. ISSN: 1755-0998. http://dx.doi.org/10.1111/1755-0998.13189 (June 2020).

120. The genome of the model beetle and pest Tribolium castaneum. *Nature* **452,** 949–955. ISSN: 1476-4687. http://dx.doi.org/10.1038/nature06784 (Mar. 2008).

121. Fu, Y. *et al.* The genome of the Hi5 germ cell line from Trichoplusia ni, an agricultural pest and novel model for small RNA biology. *eLife* **7.** ISSN: 2050-084X. http://dx.doi.org/10.7554/eLife.31628 (Jan. 2018).

122. Rodriguez-Caro, L., Fenner, J., Benson, C., Van Belleghem, S. M. & Counterman, B. A. Genome Assembly of the Dogface Butterfly Zerene cesonia. *Genome Biology and Evolution* **12** (ed Eyre-Walker, A.) 3580–3585. ISSN: 1759-6653. http://dx.doi.org/10.1093/gbe/evz254 (Nov. 2019).
